# Machine Learning Models to Forecast Outcomes of Pituitary Surgery: A Systematic Review in Quality of Reporting and Current Evidence

**DOI:** 10.3390/brainsci13030495

**Published:** 2023-03-15

**Authors:** Matheus M. Rech, Leonardo de Macedo Filho, Alexandra J. White, Carlos Perez-Vega, Susan L. Samson, Kaisorn L. Chaichana, Osarenoma U. Olomu, Alfredo Quinones-Hinojosa, Joao Paulo Almeida

**Affiliations:** 1 Department of Neurosurgery, University of Caxias do Sul, Caxias do Sul 95070-560, RS, Brazil; 2 Department of Neurosurgery, Mayo Clinic Florida, Jacksonville, FL 32224, USA; 3 Department of Neurosurgery, Penn State Health, Hershey, PA 17033, USA; 4 Department of Neurosurgery, Cleveland Clinic Lerner College of Medicine of Case Western Reserve University, Cleveland, OH 44195, USA

**Keywords:** artificial intelligence, machine learning, outcomes, pituitary adenoma, adenoma, acromegaly, Cushing disease, reporting quality assessment, systematic review

## Abstract

Background: The complex nature and heterogeneity involving pituitary surgery results have increased interest in machine learning (ML) applications for prediction of outcomes over the last decade. This study aims to systematically review the characteristics of ML models involving pituitary surgery outcome prediction and assess their reporting quality. Methods: We searched the PubMed, Scopus, and Web of Knowledge databases for publications on the use of ML to predict pituitary surgery outcomes. We used the Transparent Reporting of a multivariable prediction model for Individual Prognosis Or Diagnosis (TRIPOD) to assess report quality. Our search strategy was based on the terms “artificial intelligence”, “machine learning”, and “pituitary”. Results: 20 studies were included in this review. The principal models reported in each article were post-surgical endocrine outcomes (*n* = 10), tumor management (*n* = 3), and intra- and postoperative complications (*n* = 7). Overall, the included studies adhered to a median of 65% (IQR = 60–72%) of TRIPOD criteria, ranging from 43% to 83%. The median reported AUC was 0.84 (IQR = 0.80–0.91). The most popular algorithms were support vector machine (*n* = 5) and random forest (*n* = 5). Only two studies reported external validation and adherence to any reporting guideline. Calibration methods were not reported in 15 studies. No model achieved the phase of actual clinical applicability. Conclusion: Applications of ML in the prediction of pituitary outcomes are still nascent, as evidenced by the lack of any model validated for clinical practice. Although studies have demonstrated promising results, greater transparency in model development and reporting is needed to enable their use in clinical practice. Further adherence to reporting guidelines can help increase AI’s real-world utility and improve clinical practice.

## 1. Introduction

Pituitary adenomas (PAs) comprise 10–15% of all intracranial tumors [1]. Medical management and radiation therapy are treatment options in selected cases but transsphenoidal surgery remains the primary treatment modality for most patients with symptomatic nonfunctioning and functioning pituitary tumors, with overall low rates of morbidity and mortality [2,3]. Surgical outcomes, such as disease remission, extent of resection and complications, are influenced by different factors, including tumor size and invasiveness, previous treatments and patient age and comorbidities [4,5,6,7].

Machine learning (ML) is a type of artificial intelligence (AI) that uses imputed data to generate outputs based on the learning of patterns, which has been successfully applied across different areas of medicine [8,9,10]. The increasing volume of health care data provides inputs for innovative methods of data gathering, selection and analysis [11]. ML is especially useful in these settings because of its capacity to deal with large swaths of data [12].

ML models have shown promising results in neurosurgery. For example, ML-based imaging analysis is promising for radiological identification of glioblastoma molecular subtypes [13]; also, ML models have been used to predict outcomes of radiosurgery for cerebral arteriovenous malformations [14], and outcomes of chronic subdural hematoma [15]. Therefore, ML holds promise as a tool to augment clinical decision making [16]. Recent studies on pituitary adenomas and transsphenoidal surgery have also explored methodological designs based on ML models. Radiological diagnosis, prediction of clinical outcomes and complications have been evaluated with promising initial results [16,17,18]. Table 1 presents a glossary with the most common terms from literature and, in Table 2, we described the most common ML algorithms used in healthcare.

The popularization of studies based on AI methodology led to development of guidelines to specifically address such reports in medicine [19,20]. A version of the Transparent Reporting for Individual Prognosis Or Diagnosis (TRIPOD) Statement with focus on ML-based studies was recently proposed [21,22]. Goals of such guidelines include the assurance that studies are properly reported, providing information necessary for replicability, ensuring critical appraisal of ML models and improving the quality of reporting [22,23].

In the present study, we review the current evidence on the use of ML to predict outcomes after pituitary surgery. Additionally, we assess the completeness of model reporting of the reviewed papers according to the TRIPOD Statement.

## 2. Materials and Methods

This systematic review was conducted according to Preferred Reporting Items for Systematic Reviews (PRISMA) guidelines. The review protocol was registered within the International Prospective Register of Systematic Reviews (PROSPERO) database, maintained by the University of York (York, UK) (registration number CRD42021253264).

### 2.1. Literature Search and Studies Selection

The PubMed, Scopus, and Web of Science databases were searched to identify all potentially relevant studies. The following search terms were used: “((machine learning) or (artificial intelligence)) and (pituitary)”. Original articles that described using a machine learning approach to study pituitary surgery outcomes published between 1 January 2010 and 31 December 2021 were included.

Subsequently, three authors (M.M.R, A.W. and L.M.F) independently screened each article’s titles and abstracts. Disagreements were resolved through a discussion involving all three authors. For all studies deemed relevant, the full papers were reviewed.

### 2.2. Inclusion and Exclusion Criteria

During the full article review process, articles were included based on the following criteria: (1) specific focus on the development or validation of ML models for prediction; (2) specific focus of the model on predicting pituitary surgery outcomes; and (3) presented a ML model as its main prediction tool. Exclusion criteria were the following: (1) review articles; (2) other applications of artificial intelligence; and (3) studies using ML as a diagnostic tool. References from previous studies were also evaluated for the inclusion of additional studies.

### 2.3. Data Extraction

The data extraction protocol, as well as the form used to conduct it, is described in Online Resources 1. Outcomes were stratified in three categories: endocrine outcomes; tumor management or recurrence; or complications. If a study has reported more than one model or assessed different outcomes of pituitary surgery in a single publication, data extraction and stratification of this paper in the results section were performed regarding the model with the higher Area Under the receiver operating characteristics Curve (AUC).

### 2.4. Report Assessment

The TRIPOD Statement, launched in 2015, is a widely accepted EQUATOR Network guideline for prediction model reporting [21,24]. It consists of 22 items considered essential for informative reporting of prediction model studies. It was primarily developed to evaluate regression-based models, but it has also been successfully used to assess and guide the production of reports based on ML models [22]. It is important to mention, however, that differences in terminology are pointed out as one of the barriers to adherence to TRIPOD during the report of ML studies [25].

In this study, we utilized the TRIPOD Adherence Form as well as the instructions for its respective description, with two terms adjusted for the specification of ML models as suggested by Wang et al., to assess the completeness of reporting of ML prediction models [22,26].

## 3. Results

### 3.1. Study Selection

**Figure 1:**
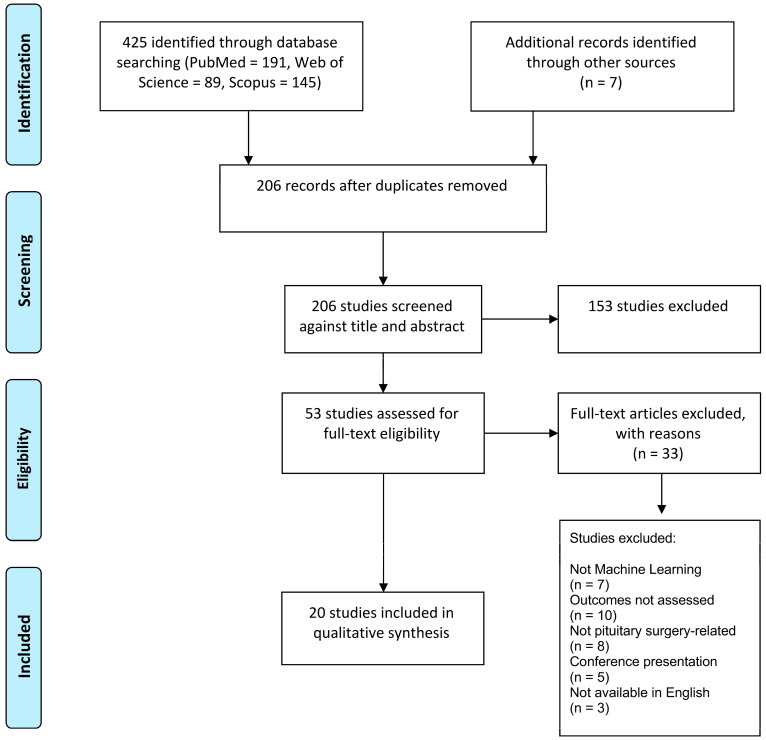
PRISMA flowchart of study search and inclusion process. PRISMA = Preferred Reporting Items for Systematic Reviews and Meta-analyses.

A total of 191 studies were retrieved from PubMed, 89 studies from Web of Science, and 145 studies from Scopus, giving a total of 425 articles. In total, 219 duplicate studies were excluded. After abstract and title screening, 53 studies were considered potentially relevant. Seven additional studies from other sources were included at this time. After full-text article screening, 20 studies were selected for data extraction (Figure 1).

### 3.2. Characteristics of Included Studies

The most common population studied were general samples of patients with PAs, without distinction (8 studies) [27,28,29,30,31,32,33,34]. Acromegaly patients were the main population in five studies [18,35,36,37,38]. Furthermore, Cushing disease (CD) patients were the focus in four additional studies [39,40,41,42]. Only two studies reported a multicenter setting for external validation [18,38]. Only Qiao et al. reported the use of a prospective sample for internal validation [18]. The time span of patient data collection ranged from 1983 [36] to 2021 [43]. Aside from Muhlestein et al. [29], which used a national inpatient administrative database, all studies gathered their data from surgeons’ case series or institutional chart review. The median sample size was 211 (IQR = 138–366) and ranged from 27 [44] to 15,487 [29]. Data extracted from the reviewed papers regarding the studies’ characteristics and ML algorithms aspects are presented in Table 3 and Table 4, respectively. Three studies had their main ML model predicting pituitary surgery outcomes regarding tumor management aspects or recurrence [27,32,44], while ten focused on the endocrine outcomes after pituitary surgery [18,35,36,39,40,41,42,43,45], and seven studies presented ML models predicting complications from pituitary surgery [28,29,30,33,34,38,46].

### 3.3. Report Assessment

**Figure 2:**
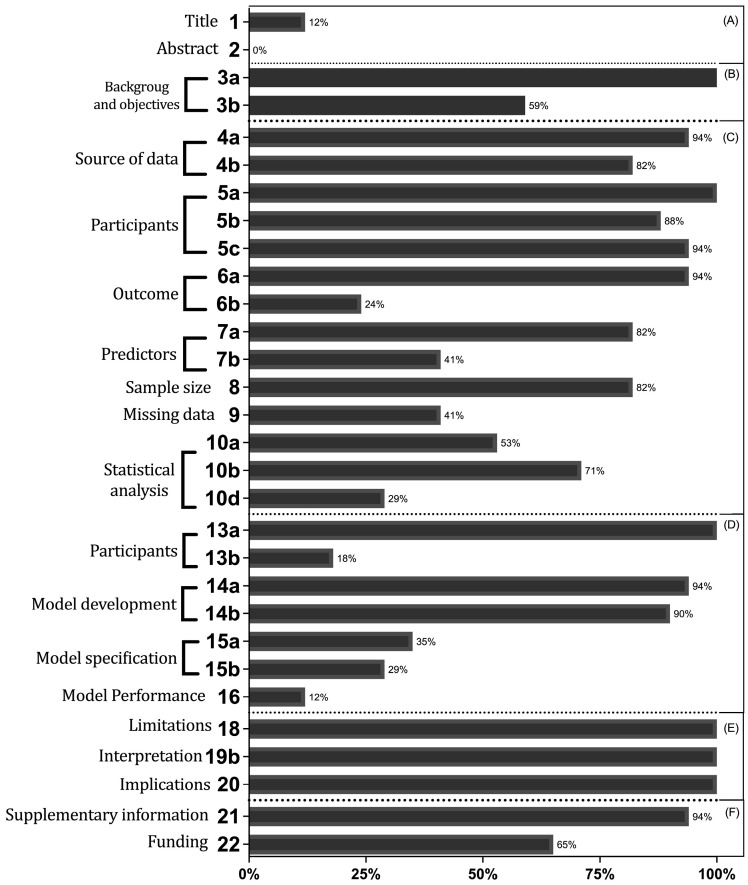
Adherence to the evaluated items and sub-items from the TRIPOD reporting standard. TRIPOD = transparent reporting of a multivariable prediction model for individual prognosis or diagnosis; A = Title and Abstract. B = Introduction. C = Methods. D = Results. E = Discussion. F = Other information.

Overall, adherence to TRIPOD among the studies had a median of 65% (IQR = 60–72%), ranging from 43% to 83. Figure 2 presents the proportions of adhered items across the included studies. The overall reporting of TRIPOD items was particularly low regarding abstract completeness of report, where no article fulfilled the criteria of the TRIPOD Adherence Form. Items concerning the report of title and performance measures (considered as adhered when discrimination with confidence intervals, calibration measure, and complementary metrics, such as accuracy, where provided) followed as the most underreported aspects—both with 12% of average adherence.

### 3.4. Models’ Assessment

**Figure 3:**
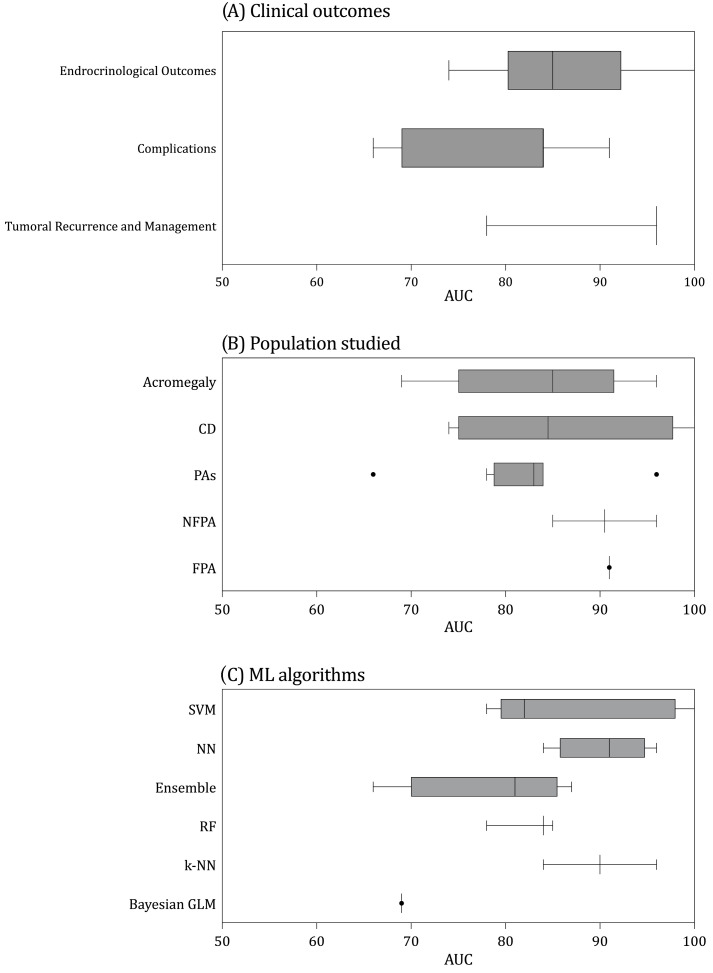
Box and whisker plots of AUC by categories of (**A**) clinical outcomes, (**B**) population studied, and (**C**) machine learning type of algorithm category. Center lines show the medians; box limits indicate the 25th and 75th percentiles; whiskers extend 1.5 times the interquartile range from the 25th and 75th percentiles, AUC values of each individual study are represented by dots. PA = Pituitary Adenoma; NFPA = Nonfunctioning Pituitary Adenoma; Functioning Pituitary Adenoma; CD = Cushing Disease; GLM = Generalized Linear Model; RF = Random Forest; SVM = Support Vector Machine; NN = Neural Network.

All models presented AUC measures to assess discrimination. The median reported AUC was 0.84 (IQR = 0.80–0.91). Figure 3 shows the AUC values reported for each of the subgroups included in this review. Moreover, calibration methods were not reported in 15 studies. When reported, the calibration methods used were the Hosmer–Lemeshow test (three studies) [36,45], calibration plot (one study) [36], calibration slope (two studies) [18,38], calibration intercept (two studies) [18,38], and the Brier Score (one study) [39].

All studies reported internal validation. The most common approach was based on k-fold cross-validation (k-CV) (11 studies) [18,27,28,32,33,37,38,40,42,43,44]. In terms of algorithm’s type, ML models derived from support vector machines (SVMs) were the most reported (five studies) [32,33,35,39,43]. They were followed by neural networks (Neural Networks) (four studies) [27,34,41,46], and Random Forest (RF) (three studies) [28,42,43]. The median AUC for SVM, NNs, and RFs was 0.82 (IQR = 0.81–0.84), 0.91 (IQR = 0.89–0.92), and 0.84 (IQR = 0.81–0.85).

### 3.5. Clinical Outcomes Predicted

#### 3.5.1. Tumor Management and Recurrence

Two studies assessed tumor recurrence as the main outcome [32,44]. Both studies used only radiomics features to build the models. AUCs were of 0.78 [32] and 0.96 [44]; however, confidence intervals were not reported for these measures. The sample size among these studies ranged from 27 [44] to 50 [32]. Only one study reported how hyperparameters were defined [44]. Both models used k-fold CV approach for internal validation. Neither study reported calibration measures. Both studies were conducted in patients with non-functioning pituitary adenomas (NFPA).

The use of radiomics approaches was prominent among studies predicting management and recurrence of pituitary tumors, exclusively inputting raw imaging data [32,44]. Zhang et al. described three important features extracted from preoperative MRI and selected by an SVM classifier to compose their ML model to predict post-surgery recurrence in NFPA [32]. Machado et al. also evaluated the prognostic value of MRI radiomics in an ML model to predict recurrence of NFPA after surgery [44]. The most important features, selected by a k-NN algorithm, to integrate the model were related to parameters of energy, total-energy, and non-uniformity, which cannot be detected by the naked-eye but represent valuable information to be accessed for prediction purposes [44]. Gross-total resection (GTR) of tumor after pituitary surgery was the outcome predicted in one study [27] and presented as a secondary outcome in two other studies [38,39] based on structured information (i.e., tabular/spreadsheet data). The algorithms utilized were NN [27], k-NN [39], and generalized linear model (GLM) [38]. Staartjes and colleagues presented a polarity correlation plot, and found that GTR was prominently correlated with the Knosp grade and the ratio between the maximum adenoma diameter and the intracarotid distance in C4 horizontal segment [27].

Regarding clinical variables, Zhang et al. found that visual disturbance, extrasellar extension, hypopituitarism, and symptoms of sexual hormones were related to persistent/recurrent disease in NFPA [33]. Furthermore, prior surgery was the most important predictor of GTR, while age and Hardy grading were predictors of biochemical remission and cerebrospinal fluid (CSF) leak, respectively, in a study by Zanier et al. [38].

AUCs values were 0.96, 0.98, and 0.68, respectively. Sample sizes were of 140 [27], 151 [39] and 307 [38] participants. Two of the studies used a k-fold CV [27,38] and the other performed a random split sample to obtain an internal validation group [39]. Calibration was reported by two of the studies (Brier Score [39], calibration slope [38], and calibration intercept [38]). Two studies reported the method to handle missing values (single imputation predictive mean matching [27] and k-NN [38]), although neither reported the missingness distribution across features. Confidence intervals were reported by two of the articles [27,38]. The approach to define hyperparameters was mentioned in one of the studies [39].

#### 3.5.2. Endocrine Outcomes

Ten studies proposed models to predict endocrinological outcomes after pituitary surgery [18,35,36,37,39,40,41,43,45]. Two studies presented models based exclusively on radiomic features [35,45]. Median AUC was 0.85 (IQR = 0.81–0.91). Sample size ranged from 47 [37] to 1045 [40] patients with a median sample size of 219 (IQR = 151–668). Five studies reported confidence intervals of their respective AUCs [18,40,41,42,45].

Definition of endocrine outcomes varied across studies. Acromegaly remission was considered off-medication GH levels (nadir GH < 0.4 µg/L during an oral glucose tolerance test, and/or random GH < 1.0 µg/L) or normalized IGF-1 (<1) at 6-month follow-up after surgery by Qiao et al. to forecast response of functioning pituitary adenomas (FPA) to surgery [18]. Fan et al. defined the endocrine outcome, postoperative remission of GH-secreting FPAs, as random serum GH < 1 ng/mL or a GH nadir < 0.4 ng/mL during an oral glucose tolerance test at 12 weeks after surgical treatment [45]. Two studies investigated CD remission, defining it as morning serum cortisol values falling below 5 μg/dL (138 nmol/L) or 24 hUFC levels falling below 20 μg (56 nmol) in the 7-day postoperative follow-ups [40,42]. Zoli et al. defined CD postsurgical remission as demonstrated hypersecretion normalization at 1 to 3–6 months after surgery (the first surgery in case of repeated procedures) [39]. Kocak et al. defined response to somatostatin analogues (SAs) in acromegaly after surgery considering patients resistant if GH or age-adjusted IGF-1 levels were still elevated after 6 months of therapy with octreotide (40 mg per 28 days) or lanreotide (120 mg per 28 days) [37]. Finally, Nadezhdina et al. defined their endpoint, CD recurrence, as one of the following: increased evening salivary cortisol level; no suppression of serum cortisol below 50 nmol/L (1.8 μg/dL) during the 1-mg dexamethasone suppression test; increased 24 h urine free cortisol level; increased concentrations and abnormal secretory rhythms of ACTH and cortisol; or clinical recurrence of hypercorticism [41].

Tumor invasiveness, usually presented using Knosp grade, was reported as being among the top three most important variables in the majority of the studies on endocrinological outcomes [35,36,39,40,45]. Tumor size was also of main importance for two studies [39,40]. The post-operative levels of GH were the second most cited among the main important variables reported in the studies [18,35,36]. In addition, ACTH and cortisol were among the most important variables of one study [42].

Regarding clinical variables, Fan et al. found that age, hypertension, ophthalmic disorders, IGF-1, elevated GH, Knosp grade and maximal tumor diameter were associated with endocrine response after surgery in patients with acromegaly [36]. In patients with CD, Zhang et al. found the highest AUC with four variables including cavernous sinus invasion in MRI, first operation, preoperative ACTH, and tumor size [40]; in another study by Liu et al., top predictors for recurrence in this subset of patients were post-operative morning serum cortisol and ACTH nadir, and age [42]. The relevance of cortisol and ACTH levels in prediction models was also confirmed by Nadezhdina et al. [41].

Four papers presented models developed on acromegaly patients [18,35,36,37], with four studying Cushing disease (CD) patients [39,40,41,42], one studying functional pituitary adenoma (FPA) patients [46], and one studying NFPA [43]. Calibration methods were reported in five studies [18,35,36,39,45]. Approaches to handle missing data were complete case analysis (one study) [41], imputation of variable median (one study) [42] and k-NN imputation (two studies) [36,40]; five articles did not report handling of missing data [18,35,37,39,45]. Methods used for defining optimal hyperparameters were reported in seven studies [35,36,39,40,42,43,45]. For internal validation, five studies reported k-fold CV (five-fold and ten-fold) [18,37,42,43,45], and one study reported leave-one-out CV (LOOCV) [35]. One study performed an external validation in a sample of 52 patients and achieved an AUC of 0.87 [18]. The median of completeness of the TRIPOD was 71.9% (IQR = 64–78%).

#### 3.5.3. Intra- and Post-Operative Complications

Seven studies presented models to predict complications during or after pituitary surgery [28,29,30,33,34,38,46]. Median AUC value was 0.84 (IQR = 0.75–0.84). The sample size ranged from 131 [33] to 15,487 [29] and presented a median of 348 (IQR = 207–400) patients. Confidence intervals were reported in four studies [28,29,34,46], although Hollon et al. provided them for accuracy instead for AUC [28]. 

Two studies adopted broad criteria defining early complications from pituitary surgery, aiming to predict at least one among a list of several events [28,29]. One of these analyzed more than 15 potential complications—e.g., extended length of stay or stroke—and presented as most influential in their model the disturbances of sodium, age, and body mass index (BMI) [28]. Muhlestein et al. proposed the prediction of any complication as a secondary analysis, aiming primarily to predict hospitals’ total charges in an administrative dataset of almost 15,000 patients [29]. Their model revealed that age, fluid or electrolyte abnormalities, and admission type were the most important variables to predict complications in that sample [29]. 

Staartjes et al. proposed a ML model to estimate risk of intraoperative CSF leakage using an NN algorithm [34]. They reported a high suprasellar Hardy grade, prior transsphenoidal surgery, and age as contributing most to the outcome prediction [34]. In an effort to predict suboptimal outcomes—defined as hormonal non-remission or MRI evidence of recurrence/progression despite adjuvant treatment—Shahrestani et al. built an NN model and inputted clinical variables that were significant in a multivariate statistical analysis [46]. The authors found that additional surgery, preoperative visual deficit not improved after surgical intervention, and transient diabetes insipidus increased the odds of suboptimal outcomes [34].

Five studies reported methods to handle missing values. The models were developed on general samples of PAs patients (four studies) [28,29,30,34], on a sample of mixed types of FPAs [46], and on a sample of acromegaly patients [38]. Methods for selection of hyperparameters were reported by three studies [28,30,34]. Calibration techniques were mentioned by one of these studies (calibration slope and calibration intercept) [38]. The median of completeness of the TRIPOD was 62.1% (IQR = 52–63%).

## 4. Discussion

This systematic review addressed the quality and breadth of studies using ML methodology to predict outcomes of pituitary surgery. Heterogeneity in model reporting may impact the full understanding of ML’s role in outcome prediction for patients with pituitary tumors and makes it challenging to conduct a meta-analysis of existing studies. Nonetheless, interest in the topic has substantially increased in the last decade, which highlights the importance of adequate reporting to maximize the usefulness of this approach in clinical research and patient care.

### 4.1. Clinical Findings

Regarding prediction of pituitary surgery outcomes by ML methods, an important part to ensure its use in clinical practice relies on variable importance analysis. In this review, aspects of tumor invasiveness were mentioned among the top predictors in the majority of the studies, regardless of the classifying system adopted (Table 5). These results agree with a previous review which found that cavernous sinus invasion is the best single predictor of tumor remission [48]. Knosp grade is also mentioned as a good predictor for GTR in previous studies [49,50]. Despite the existence of other tumor invasiveness scales, such as the Hardy Grade, these are less used in the actual clinical context [51]. Nevertheless, those tools present limitations such as allocating patients into large groups of risk and not tailoring individual characteristics, as well as problems in poor interrater reliability [52]. 

In additional to measures of invasiveness, endocrinological parameters integrated most of the models (Table 5). Age was the most common demographic variable utilized in the models and was the one demographic with high importance reported across different studies (Table 5). Externally validated ML algorithms can play a major role in precise risk stratification and in identifying patients who will not likely benefit from surgery or adjuvant therapy [16,49].

Furthermore, the analysis of clinical images through ML algorithms is prominent in ML models to predict pituitary surgery outcomes (Table 5). ML algorithms are trained to mine quantitative imaging features from medical images, looking for patterns between the images and outcome of interest [53,54]. Fan et al. and Niu et al. presented a direct comparison of their results using ML models inputted with radiomics and clinical features against the predictive power of Knosp grade alone [35,55]. In both cases, the ML-based approaches overperformed the traditional tool. Indeed, the studies from our review that combined radiomic signatures with clinical features and other types of structured data presented better performance than both forms—radiomics or structured data—alone. 

The open-source availability of any reported model is a good practice in research and contributes to transparency as well as to the presentation of the real value of the developed model for clinical practice. The description of nomograms is one of the forms to make a model useful and valuable in practice. In our review, nomograms were presented in two papers, both carried out by Fan et al. [35,45]. In one of them, the authors presented a nomogram that uses the radiomic signatures obtained using the ML algorithm and the Knosp grade [45]. In the other study, the nomogram was composed of radiomics signature, random GH, IGF-1 standard deviation score, GH inhibition ratio, tumor volume, Knosp grade, tumor consistency, and P53 value [35]. Three studies provided access to their models, deploying them as web-based clinical calculators: Qiao et al. for predicting post-surgical acromegaly remission based on demographics, tumor characteristics and hormone levels (https://deepvep.shinyapps.io/Acropred/, accessed on 1 December 2022); Zhang et al. to predict immediate remission of histology-positive CD patients after surgery (http://smk921101.pythonanywhere.com/index, accessed on 1 December 2022); and Nadezhdina et al. for the prediction of recurrence and remission within 3 years in patients with Cushing (https://medcalc.appspot.com/eng_ver, accessed on 1 December 2022) [18,40,41].

### 4.2. Report Assessment

As measured by the TRIPOD, the rates of report completeness were suboptimal for several items of the overall assessment. However, certain TRIPOD items are significantly more important to ensure research utility and quality than others. For instance, although only one article showed completeness of reporting in the Title and Abstract—Items 1 and 2, respectively—the lack of information on how missing data was handled and how the models were calibrated has a greater impact on reviewers’ ability to assess the quality of these studies.

Calibration measures were reported by only three studies, which demonstrates a potential for improvement in future projects. Calibration is used to assess reliability of risk predictions of a given model. Thus, a good calibration implies predicting an event for a person with a specific feature matching with the proportion of all people in the population with similar feature values who experienced the event [11]. Therefore, even with a good discriminative performance described by AUC, it is not enough to provide a critical appraisal of the model and, consequently, not enough to properly guide clinical decisions. To make this possible, both a discrimination (e.g., AUC) and a calibration measure (e.g., Brier score) should be presented [56]. The lack of information about the latter can imply misinterpretation of a given ML model, lower clinical usefulness, compromising potential external validation by others, and unnecessary risk to patients.

Information on how hyperparameters of the final models were defined was mentioned in 10 studies [28,30,34,35,36,39,40,42,44,45]. Hyperparameters settings significantly interfere in the final performance of the prediction model [57]. The most common approach utilized in the studies for hyperparameters selection was Grid Search CV—a method that iteratively tests all potential values for hyperparameters, choosing the ones with the result in the higher values of the metric of interest (e.g., AUC, F1-Score or accuracy)—which is also the method most commonly reported in the literature, although it is not always an ideal choice, given the chances of overfitting training datasets [58]. In addition, even the same model algorithm often needs different hyperparameter settings when training on different datasets during out-of-sample validations. For instance, in deep learning (DL) models, hyperparameters such as the number of layers or the dropout rate can dramatically affect performance in a NN algorithm [57,58]. Publishing the algorithm code, including exact hyperparameters utilized, allows for a rigorous assessment of the model and prevents redundant research from being undertaken.

Only two studies presented external validation [18,38]. External data are significantly important to assess real-world performance since they can measure performance losses and provide insight about biases in some step of the model’s development. External validation is recommended to be performed at a different time (temporal validation) or location (geographical validation) from the original dataset which derived the initial ML model. Every model with only internal validation is marked by the idiosyncrasies of the original population and may thus perform poorly in others. This is true for a wide range of factors, including changes in policies, practice and demographics [59,60]. Methods for handling missing values were fully reported by three of the studies [29,42,46]. When the handling of missing data was mentioned but not fully reported, it was usually due to not reporting the number of missing values, the variable where the imputation was performed or the number of imputations, an important factor for the reliability of a model. However, from the studies that explicitly described the method used to replace the missing data, only 10 reported the used approach satisfactorily [27,28,29,30,34,36,40,41,42,46]. When data are considered missing at random, multiple variable imputations, they are usually considered superior to single imputation and complete case analysis by preserving the natural variability of the missing values, and retains more useful information, respectively [61,62]. Within our results, only one study reported a form of multiple imputation [46].

### 4.3. ML versus Traditional Statistical Methods

Despite the exponential growth of AI research in medical areas during the last two decades, the real advantage of the use of ML over traditional statistical methods such as regression analyses remains under question. A systematic review conducted by Christodoulou et al. showed that discriminative measures of ML models to predict clinical risk compared with logistic regression were significantly higher only in comparisons with a high risk of bias and similar in the comparisons with low risk [63]. A common rationale for the development of ML models among the studies reviewed above was the capability of ML to identify and handle nonlinear interactions, which traditional methods would not perform so well with. Other authors report unsupervised ML’s potential to analyze large, unorganized, and highly complex amounts of information, channeling the potential of big data to create prediction models [64].

There is more evidence for outperformance by ML compared to traditional models in neurosurgery, as reviewed by Azimi et al. regarding applications of NNs [65]. Specifically, this advantage was also reported in studies about pituitary-related ML applications [17,47]. When reporting the performance of prediction models on sellar diseases, Qiao reported a higher predictive power of ML algorithms compared to conventional regression methods but acknowledges concerns about the models such as the fact that ML methods are more time- and data-consuming compared to traditional statistics and less effective in several cases [47].

Another important difference between ML and traditional statistics lies in the interpretability of each predictor and the interpretability of the final model. While traditional statistics can offer concrete mathematical rationales between inputs and outputs and consequently optimal explicability, ML is often labeled as a “black box”. That is, even with plain knowledge about all model’s inputs and outputs, the generalization of the internal decision-making process is not feasible. Some authors described this phenomenon as a trade-off between performance and explicability, where one important aspect is sacrificed to obtain an optimal outcome in the other, also relevant [66]. In 2018, the European Union pioneered inserting in its General Data Protection Regulation that “meaningful information about the logic involved” in all decisions made by artificially intelligent systems should be provided [67]. This “right to explanation” has grounded a movement in favor of explainable AI models, which advocates that even with extremally high metrics, when choosing between models with inherent complexity and more simple ones, (e.g., Decision Trees or Random Forest) that provides interpretability, the latter should be taken [68].

Some solutions have been proposed to solve the explicability issue in ML. An innovative form for assessing variables’ importance robustly and which reached wide use recently is the Shapley additive explanation (SHAP) approach, reported as an explainer for ML models by Lundberg and colleagues [69]. Originally developed in the context of game theory as a form to look after theoretically optimal solutions for cooperative games, SHAP values can be used to assign quantitative distributions of the total risk to individual model features. In brief, SHAP values apply cooperative game theory concepts to assign theoretically optimized distributions of the total risk of a given outcome to the individual model features [70]. In game theory, this is analogous to assigning each player on a team a ranked value for their contributions towards the team’s overall outcome. Nevertheless, even with potential solutions to the interpretability issue inherent to ML, there is no current consensus about a reliable metric or tool to assess the quality or accuracy of these explanations [68].

### 4.4. ML-Specific Reporting Guidelines

It is expected that a best practices culture regarding all the steps towards ML models’ clinical implementation will be promoted and encouraged by adherence to ML-specific protocols and statements. To illustrate the guidelines’ potential, clinical trials’ reporting had a significant improvement in quality after the release of CONSORT and SPIRIT, particularly when the adherence to them started to be mandatory amongst peer-reviewed journals [71,72]. Moreover, a crucial milestone to successfully implementing “good practices on ML modelling” also depends on establishing those proper standards as a mandatory requirement to further ML-model publication by peer-reviewed journals in the medical area.

### 4.5. Future Perspectives

To date, pituitary surgery has received less exploration than other neurosurgical entities regarding ML modeling. Other potentially relevant approaches may be pursued, particularly concerning the use of radiomics as a part of the development of new algorithms. Innovative applications such as the use of intraoperative MRI may present a pathway to clinical significance. Particular subjects, e.g., acromegaly condition, may benefit from future original studies and reviews scrutinizing surgical outcomes predictions and aspects such as diagnosis (e.g., facial recognition) or response to medical therapy.

### 4.6. Strengths and Limitations

This systematic review has inherent limitations. First, the data are substantially heterogeneous across the studies, limiting further comparison between the studies or meta-analytic approaches. Second, this review focused only on ML models predicting pituitary surgery outcomes and analyzed the quality of report of the respective studies. Thus, our review cannot comment on the performance of traditional statistical methods. Overall, evidence is limited by the lack of transparency in the reporting of the studies. This scope of literature could also benefit from a formal assessment of the risk of bias of published studies, for example, with the use of PROBAST (Prediction model Risk Of Bias Assessment Tool) [73]. The use of TRIPOD-AI guidelines may facilitate a more comprehensive reporting of ML model development methods in future publications. This review also has several strengths. Firstly, the review was performed under two guidelines: PRISMA checklist and TRIPOD Adherence Form, aiming for consistency and transparency. We provided the rationales and importance of some of the most poorly reported items in the TRIPOD which could enhance and provide insight for further reviews, as well as for future development and validation of ML models. To the best of our knowledge, this is the first systematic review to include assessment of report completeness in regard to ML in neurosurgery. Finally, this review provides a comprehensive account of the use of ML methods to predict patient outcomes after pituitary surgery.

## 5. Conclusions

Applications of ML in the prediction of pituitary outcomes are still nascent. Even though the articles presented in this review have a broad range of applications on pituitary surgery, current data suggest that there is an area of opportunity for improving the quality of ML model reporting. The use of report guidelines should be encouraged mainly by peer-reviewed journals. The release of TRIPOD-AI is expected to address this need and contribute to ML research applied to healthcare predictions.

## Figures and Tables

**Table 1:** Definitions of important concepts in machine learning and artificial intelligence areas.

Term	Description
Artificial Intelligence	A broad area of computer applications with the ability to perform tasks that conventionally require human intelligence.
Machine Learning	Machine learning is an application of artificial intelligence (AI) that provides systems with the ability to automatically learn and improve from experience without being explicitly programmed.
Deep Learning	Is a subset of ML which is formally defined as computational models that are composed of multiple processing layers to learn representations of data with multiple levels of abstraction.
Supervised learning	A model that is trained based on inputs of data aiming at determining a target output, which are manually labeled a priori (e.g., diagnosis or prognosis).
Unsupervised learning	ML models that can perform tasks without being set with labels by a human (e.g., clustering data).
Structured data	Data that are pre-defined to be displayed in rows and columns (e.g., electronic medical records, administrative data). More qualitative form of data.
Unstructured data	Data without any predefined structure. More quantitative form of data (e.g., image analysis, text).
Missing values	Hyperparameters, which specify how a model learns, need to be set by the data scientist before training. They are perpetually improved (tuned) to find the model that performs best.
Single case analysis	Exclusion of a row with missing data among its features.
Feature	Data science term for predictor/independent variable.
Label/Target	Data science term for outcome/dependent variable.
Parameter	Inherent weights of a given model, which are set in the code of the algorithm. Define a search space as a grid of hyperparameter values and evaluate every position in the grid.
Hyperparameters	An ensemble of wights which define how a model learns. They are arbitrarily attributed, needing to be set by its developer to optimize its performance during and after training.
Overfitting	When a model performs well on the training data (seen patients) and performs poorly on the testing data (unseen patients). Regularization is often used to minimize overfitting and optimize generalizability of machine leaning algorithms
Discrimination	Describes the model’s ability to correctly identify from random pairs in which it was trained who will develop the target condition. Usually evaluated through the model’s AUC/C-statistic.
Area Under Curve/C-statistic	Most used discriminative statistic. An area of 1.0 represents a perfect test; an area of 0.5 represents a worthless test. It enables assessment of predictive ability, and identification of an optimal threshold to distinguish between classes.
Accuracy	Ratio between the total number of predictions that are correct.
Sensitivity/Recall	Proportion of true positives predictions.
Specificity	Proportion of correctly predicted true negatives which are correctly identified.
PPV/Precision	Proportion of correctly predicted true positives which are correctly identified
NPV	Proportion of correctly predicted negatives among all negative predictions.
F1 score	Composite metric defined as the harmonic mean between precision (or PPV) and recall (sensitivity).
Internal Validation	Assessment of a model’s performance with the same data or population, if prospective, used in the development process.
External Validation	Assessment of a model’s performance in a dataset which differs from the one used in its development geographically or temporally.
Cross Validation	Internal validation technique in which the dataset is randomly split into k-1 groups of similar size. Performance is evaluated in the remaining group with the whole process repeated n times; model performance is taken as average over n iterations.
Bootstrapping	Internal validation approach like cross validation but relying on random sampling with replacement; each sample is the same size as model development dataset
Split Sample	Internal validation approach in which the available development dataset is divided into two datasets: one to develop the model and the other to validate the model; division can be random or non-random.

**Table 2:** Examples and conceptualization of most utilized machine learning-based algorithms for binarity outcome prediction.

Algorithm	Description
Neural Networks (NN or ANN)	Artificial neural networks are non-linear algorithms loosely inspired by human brain synapses. Convolutional neural networks, the most commonly applied, comprise input nodes, output nodes and intervening or hidden layers of nodes, which may number up to 100. Each node within a layer involves two or more inputs and applies an activation and weighting function to produce an output which serves as the input data for the next layer of nodes.
Support Vector Machine (SVM)	SVM is based on the idea of computing a hyperplane that best separates the features into different domains. Its objective is to find a decision boundary (the Hyperplane) that has the maximum separation degree between two nearer points of each class—i.e., the support vectors. Kernel functions are used when data are too non-linear functions; the algorithm can map examples to other dimensions and then operates on non-linear relationships by transforming low-dimensional input data into high-dimensional space.
k-Nearest Neighbors (k-NN)	The k-NN classing classes based on a distance criterion. The values of the distance from k (number of neighbors) in given distance between them and the subject of interest. This distance inputs-output is computed on comparing multidimensional vectors of feature values, defining the more similar ones as “neighbors”.
Decision Trees (DTs)	DT algorithms are architecture under a tree structure modeling approach with conditional control statements for establishing a framework of subsequent decisions. Its internal nodes represent ‘test’ on an attribute, branch represents the results of this test and “leaf” represents decision taken after computing all attributes.
Random Forest (RF)	RF is essentially an ensemble of DTs, although it differs from usual DTs by using randomly selected inputs or combinations of inputs at each node to grow each tree rather than a consistent set. That is intent yielding to avoid the overfitting usually present in deep DTs. Random distribution of inputs provides, when averaged, lower rates of error in the final output and reduced variance.

**Table 3:** Characteristics of included studies.

Study	Journal	Country	No. of Centers	Population	Time Interval	Source of Data	Sample Size
Fan et al., 2019 [35]	*European Journal of Radiology*	China	Single-Center	PAs	April 2012 May 2018	Chart review	163
Fan et al., 2019 [45]	*Frontiers in Endocrinology*	China	Single-Center	Acromegaly	January 2008 and January 2016	Case series	57
Fan et al., 2020 [36]	*Endocrine*	China	Single-Center	Acromegaly	1983 to 2018	Chart review	668
Fang et al., 2021 [43]	*Frontiers in Endocrinology*	China	Multicenter	NFPAs	2015 to 2021	Retrospective database	215
Hollon et al., 2018 [28]	*Journal of Neurosurgery*	USA	Single-Center	PAs	Not mentioned	Case series	400
Kocak et al., 2018 [37]	*European Radiology*	Istanbul	Single-Center	Acromegaly	January 2009 and December 2017	Chart review	47
Liu et al., 2019 [42]	*Neuroendocrinology*	China	Single-Center	CD	January 2000 to December 2017	Case series	354
Machado et al., 2020 [44]	*Computes in Biology and Medicine*	Brazil	Single-Center	NFPAs	Not mentioned	Electronical Clinical Records	27
Muhlestein et al., 2019 [29]	*Journal of Neurosurgery*	USA	National Inpatient Database	PAs	2002 to 2011	Administrative data	15487
Nadezhdina et al., 2019 [41]	*Pituitary*	Russia	Single-Center	CD	2007 to 2014	Chart review	219
Qiao et al., 2021 [47]	*Pituitary*	China	Multicenter	Acromegaly	2010 to 2018 (D); 2019 (EV)	Prospective database	833 (D); 52 (EV)
Shahrestani et al., 2021 [46]	*Pituitary*	USA	Single-Center	FPA	1992 to 2019	Chart review	348
Staartjes et al., 2018 [27]	*Neurosurgical Focus*	Switzerland	Single-Center	PAs	October 2012 onwards	Prospective clinical registry	140
Staartjes, et al., 2019 [34]	*Journal of Neurosurgery*	Switzerland	Single-Center	PAs	October 2012 onwards	Prospective clinical registry	154
Voglis et al., 2019 [30]	*Pituitary*	Switzerland	Single-Center	PAs	October 2012 to December 2019	Case Series	207
Zanier et al., 2021 [38]	*Endocrine*	Switzerland	Multicenter	Acromegaly	August 1998 to January 2020	Chart review	307 (D); 40 (E)
Zhang et al., 2020 * [32]	*Frontiers in Oncology*	China	Single-Center	NFPAs	September 2010 to December 2017	Chart review	50
Zhang et al., 2021 * [33]	*Frontiers in Endocrinology*	China	Single-Center	CD	February 2000 and September 2019	Chart review	1045
Zhang et al., 2021 [40]	*Journal of Personalized Medicine*	China	Single-Centre	PAs	January 2017 to June 2019	Chart review	131
Zoli et al., 2020 [39]	*Neurosurgical Focus*	Italy	Single-Center	CD	May 1998 to December 2017	Case series	151

Abbreviations: CD = Cushing Disease; PAs = Pituitary Adenomas, NFPA = Non-Functioning Pituitary Adenoma; D = Development; EV = External Validation; * studies that only used radiomics.

**Table 4:** Machine learning models characteristics.

Study	ML Task	Outcome; Proportion	Software	Algorithm	AUC	Other Measures
Fan et al., 2019 [35]	Treatment response	Remission; 66 patients (33.7%)	MATLAB 2015b (Natick, MA, USA)	SVM	0.81	Acc: 74.5%; Sn: 61.3%; Sp 91.7%; PPV: 70.5%; NPV: 64.7%
Fan et al., 2019 [45]	Radiotherapeutic response	radiotherapy response; 25 patients (78.1%)	ITK-SNAP 3.8 (Philadelphia, PA, USA); Python 3.0 (Wilmington, NC, USA), PyRadiomic library	SVM	0.96	Acc: 91%; Sn: 90%; Sp: 92%; PPV: 935; NPV 0.885
Fan et al., 2020 [36]	Remission of acromegaly after surgery	Acromegaly remission; 349 patients (52.2%)	Python 2.7 (Wilmington, NC, USA)	GBDT	0.81	Acc: 79%; Sn: 81%; Sn: 78%; PPV: 81%; NPV: 77%
Fang et al., 2021 [43]	Postoperative hypofunction, new postoperative hypofunction, and hormonal recovery	hormone level normalization; 21 patients (64.7%)	R 4.0.4 (Vienna, Austria); Python 3.9 (Wilmington, NC, USA)	RF	0.85	AUC-PR: 0.52
Hollon et al., 2018 [28]	Poor early postoperative outcome	Poor early postoperative outcome; 124 (31%)	R 4.0.4 (Vienna, Austria), caret package; Python 3.2 (Wilmington, NC, USA), SciPy 0.19.1 library	RF	0.84	Acc: 85%; Sn: 56%; Sp: 94.7%; PPV: 77.8%; NPV: 86.6%
Kocak et al., 2018 [37]	Response to somatostatin analogues	Responsive; 24 patients responsive (51%)	WEKA 3.8.2 (Waikato, New Zeland)	k-NN	0.85	Acc: 85.1%
Liu et al., 2019 [42]	Immediate CD remission	CD recurrence; 46 patients (13.0%)	Python 2.7 (Wilmington, NC, USA)	RF	0.78	Acc: 87%; Sn: 71.7%; Sp: 58.4%
Machado et al., 2020 [44]	Tumor recurrence	Tumor recurrence; 10 (37%)	Python 3.0 (Wilmington, NC, USA), Scikit-learn library	k-NN	0.96	Acc: 96.3%; Sp: 100%; Sn: 91.7%
Muhlestein et al., 2019 [29]	Hospital total charges, Postoperative complications	Postoperative complications; 3365 inpatients (25%)	Python 2.7 (Wilmington, NC, USA), SciPy 0.17 library; DataRobot 3.0 (Boston, MA, USA)	GBDT	0.66	RMSLE: 0.446; Holdout: 0.455
Nadezhdina et al., 2019 [41]	Remission/Recurrence of CD	Remission; 172 patients (78.5%)	IBM SPSS 18 (Armonk, NY, USA)	NN	0.91	Acc: 92%; Sn: 75%; Sp 97%; PPV: 85%; NPV: 93%
Qiao et al., 2021 [47]	Acromegaly endocrine remission	Remission; 434 patients (52.1%)	R version 3.4.3 (Vienna, Austria); Python version 3.6 (Wilmington, NC, USA)	GBM	0.87	Acc: 80.3%; Sn 90.5%; Sp 69.6%
Shahrestani et al., 2021 [46]	Suboptimal outcomes	Suboptimal outcomes; 81 patients (23.3%)	Python 2.7 (Wilmington, NC, USA), PyRadiomics, Scikit-learn libraries	NN	0.91	Acc: 87.1%; Sn: 89.5%; Sp: 76.9%; PPV: 94.4%; NPV: 62.5%
Staartjes et al., 2018 [27]	GTR	GTR; 95 patients (68%)	R 3.4.4 (Vienna, Austria), TensorFlow, Keras	NN	0.96	Acc: 91%; Sn: 94%; Sp 89%
Staartjes, et al., 2019 [34]	Risk level of intraoperative CSF Leak	CSF leak; 45 patients (29%)	R 3.5.1 (Vienna, Austria); TensorFlow (Mountain View, CA, USA), Keras	NN	0.84	Sn: 83%; Sp: 89%; PPV: 71%; NPV: 94%; F1 score: 0.77
Voglis et al., 2019 [30]	Post-operative hyponatremia	Post-operative hyponatremia; 44 patients (22%)	R 3.6.2 (Vienna, Austria), caret package	Boosted GLM	0.84	Acc:78.4%; Sn: 81.4%; Sp: 77.5%; F1 Score: 62.1%; NPV: 93.9%; PPV: 50%
Zanier et al., 2021 [38]	GTR, Biochemical remission, or CSF leak	CSF leak; 38 patients (12.5%)	R 4.0.2 (Vienna, Austria)	Bayesian GLM	0.69	Acc: 60%; Sn: 71%; Sp: 59%; PPV: 19%; 93%; Calibration intercept: −1.77; calibration slope: 0.39
Zhang et al., 2020 * [32]	NFPA recurrence	Tumor recurrence; 28 patients (56%)	MATLAB 2018b (Natick, MA, USA)	SVM	0.78	Acc: 82%
Zhang et al., 2021 * [33]	Postoperative Immediate Remission of CD	CD remission; 766 patients (73.3%)	R Studio 1.2 (Vienna, Austria); IBM SPSS 23 (Armonk, NY, USA); Python 3.6 (Wilmington, NC, USA), Scikit-learn library	Stacking	0.74	Acc: 72%
Zhang et al., 2021 [40]	Visual field recovery following pituitary adenoma surgery	Visual field recovery; 79 patients (60.3%)	ITK-SNAP (Philadelphia, PA, USA); R 3.6.3 (Vienna, Austria)	SVM	0.82	Acc: 70%; Sn: 65%; Sp: 80%; PPV: 70%; NPV: 80%
Zoli et al., 2020 [39]	GTR, postsurgical remission, and long-term control of disease	GTR; 137 patients (91%)	R 3.5.2 (Vienna, Austria)	SVM	1.00	Acc: 100%; Sn: 100%; Sp: 100%; PPV: 100%; NPV: 100%; F1 score: 100%; Brier score: 0.097

CD = Cushing Disease; GTR = Gross-Total Resection; CSF = Cerebrospinal Fluid; NFPA = Non-Functioning Pituitary Adenoma; SVM = Support Vector Machine; GBDT = Gradient Boosting Decision Tree; RF = Random Forest; k-NN = k-Nearest Neighbors; GLMboost = Generalized Linear Model Boost; Acc = Accuracy; Sn = Sensitivity; Sp = Specificity; PPV = Predictive Positive Value; NPV = Negative Predictive Value; RMSLE = Root Mean Squared Logarithmic Error; * studies that only used radiomics.

**Table 5:** Tabulated data utilized as input into the ML models.

Study	Demographics	Medical History	Tumor Morphology and Behavior	Endocrine Parameters	Surgical Aspects	Histological
Fan et al., 2019 [35]	Age; sex	NA	Diagnosis type of tumor; KG	NA	NA	NA
Fan et al., 2019 [45]	NA	NA	KG; tumor consistency; tumor volume;	Random GH; IGF-1 standard deviation score; GH inhibition ratio;	NA	P53
Fan et al., 2020 [36]	Age	Hypertension, ophthalmic disorders, maximal tumor diameter	KG	GH, IGF-1, nadir GH,	NA	NA
Fang et al., 2021 [43]	NA	NA	NA	Preoperative hormone levels (SH, FSH, LH, PRL, ACTH	NA	NA
Hollon et al., 2018 [28]	Age; gender; race; BMI	Prior cardiovascular, renal, pulmonary or hepatic disease; prior TSS, prior craniotomy; current blood thinners intake; prior visual deficit; use of postoperative desmopressin	Tumor type	Postoperative sodium low; postoperative sodium elevated; diabetes insipidus	NA	NA
Kocak et al., 2018 [37]	NA	NA	NA	NA	NA	NA
Liu et al., 2019 [42]	Age	Disease course;	NA	Postoperative levels of morning ACTH (nadir), morning serum cortisol (nadir), 24 h UFC; preoperative levels of morning ACTH, and serum cortisol	NA	NA
Machado et al., 2020 [44]	NA	NA	NA	NA	NA	NA
Muhlestein et al., 2019 [29]	NA	NA	NA	NA	NA	NA
Nadezhdina et al., 2019 [41]	Sex; age	Duration of disease (months)	Type of tumor;	Postoperative morning levels of ACTH and cortisol	NA	NA
Qiao et al., 2021 [47]	Age; gender; BMI	TSS; specific pharmacotherapy; radiotherapy	Tumor dimensions; KG; clivus invasiveness; intraoperative cavernous sinus invasion; tumor texture; presence of pseudocapsule;	Serum random GH; serum IGF-1 level; preoperative hypopituitarism; preoperative diabetes insipidus	Surgeons’ experience (based on annual pituitary operations performed surgical approach; total resection or subtotal	NA
Shahrestani et al., 2021 [46]	NA	Hospital LOS (days); prior craniotomy; preoperative visual deficit not improved after surgery;	NA	Transient diabetes Insipidus; low cortisol axis low GH axis; panhypopituitarism; acromegaly	NA	MIB-1/Ki-67 labeling index
Staartjes et al., 2018 [27]	Sex; age	TSS	KG; HG; tumor invasiveness; ICD at the C6, C4 horizontal, and C4 vertical segments; R ratio between maximum adenoma diameter and ICD C4 horizontal segment; adenoma secretory status, volume, and diameters in 3 axes	NA	NA	NA
Staartjes, et al., 2019 [34]	Sex; age	TSS	KG; HG; tumor invasiveness; ICD at the C6, C4 horizontal, and C4 vertical segments; R ratio between maximum adenoma diameter and ICD C4 horizontal segment; adenoma secretory status, volume, and diameters in 3 axes	NA	Targeted level of resection	NA
Voglis et al., 2019 [30]	Sex; Age; weight; height; BMI	TSS;	KG; HG	Hypofunctional ACTH and GNRH in preoperative levels; prior diabetes insipidus; preoperative levels of potassium, sodium, cortisol, IGF-1, fT3, fT4, TSH, LH, FSH, and PRL	NA	NA
Zanier et al., 2021 [38]	Age; gender	TSS	KG; HG; tumoral size	NA	NA	NA
Zhang et al., 2020 * [32]	NA	NA	NA	NA	NA	NA
Zhang et al., 2021 * [33]	NA	NA	NA	NA	NA	NA
Zhang et al., 2021 [40]	NA	TSS	Cavernous sinus invasion on preoperative MRI; tumor size	Preoperative ACTH	NA	NA
Zoli et al., 2020 [39]	Age; sex	TSS; specific pharmacotherapy; radiotherapy	Tumor size; HG; KG; bony tumor or cavernous invasiveness	Preoperative hypopituitarism; preoperative diabetes insipidus	NA	NA

LOS = Length of stay; Body Mass Index = BMI; HG = Hardy Grade; KG = Knosp Grade; TSS = Transsphenoidal surgery; ICC = Intercarotid distances; UFC = urinary free cortisol; NA: Not Applicable, i.e., the respective paper did not present variables inset in the model regarding this type of data; * studies that only used radiomics.

## Data Availability

Data available on request due to privacy and ethical restrictions.
